# The Spillover Influence of Household Waste Sorting on Green Consumption Behavior by Mediation of Environmental Concern: Evidence from Rural China

**DOI:** 10.3390/ijerph17239110

**Published:** 2020-12-06

**Authors:** Huiling Wang, Ying Ma, Shaoxiong Yang, Mansoor Ahmed Koondhar, Rong Kong

**Affiliations:** 1 School of Economics and Management, Northwest A&F University, No. 3 Taicheng Road, Yangling, Xianyang 712100, China; huilingwang@nwafu.edu.cn (H.W.); maying@xsyu.edu.cn (Y.M.); sysx319@163.com (S.Y.); 2018071024@nwafu.edu.cn (M.A.K.); 2 School of Economics and Management, Xi’an Shiyou University, No. 18 Dianzi Road, Xi’an 710065, China

**Keywords:** waste sorting behavior (WSB), rural households’ green consumption behavior, environmental concern, propensity score matching method (PSM), mediating effect model

## Abstract

The spillover effect of environmental behavior has been of wide concern in recent years. The purpose of this paper is to explore the influence of household waste sorting on green consumption (behavioral spillover) and the possible psychological mechanisms involved in such spillover of environmental concern. Though it is important, insufficient attention has been paid to exploring the relationship, and the process of its formation, between waste sorting and green consumption. To narrow this gap, survey data collected in 2018 from 688 rural households from Shaanxi Province in western China were used. The propensity score matching method was employed to measure the effect of waste sorting on rural households’ green consumption. The mediating model was employed to investigate the path of influence in the relationship between waste sorting and green consumption. The results showed that waste sorting behavior positively spilled over into green consumption, with a net effect of 0.205. Environmental concern has a mediating effect on the relationship between waste sorting and green consumption behavior, with a mediating effect of 0.3177. In summary, household waste sorting behavior has a spillover effect on green consumption behavior as a result of the mediation effect of environmental concern. The results of this article fill in our knowledge on the spillover effects of waste sorting behavior in developing countries. Policy makers and regulators should vigorously advocate and implement waste sorting behavior, increase farmers’ concern for the environment, and promote their participation in green consumption behavior, so as to maximize the spillover effect.

## 1. Introduction

People have realized that industrial civilization has improved living standards; however, it has also led to a series of severe ecological problems in terms of the global environmental crisis and resource depletion [1]. Taking China as an example, the data show that China produces nearly 1 billion metric tons of waste every year, including 400 million metric tons of domestic waste, 500 million metric tons of construction waste, and 10 million metric tons of kitchen waste. The amount of waste is growing by 8% to 10% every year in China (Published by the China Association of urban environmental sanitation at http://cnues.com). Additionally, the rural population accounts for more than 45% of the total population in China, but the amount of waste reaches up to 0.86 kg per person per day, seriously restricting transformations toward environmental improvement [2].

China’s central ministries and commissions have carried out many proposals and policy deployments for waste sorting. In the “Notice on Announcement of Pilot Cities for Domestic Waste Sorting” issued by the Ministry of Construction in 2000, eight cities, including Beijing, Shanghai, Guangzhou, and Shenzhen, were designated as pilot cities for waste sorting. Since then, the State Council of China and relevant ministries and commissions have successively issued a series of relevant policy documents. The waste sorting policy was characterized by advocacy by China’s central department and the local governments responsible for promoting it. In 2017, the Ministry of Housing and Construction of China chose 100 pilot counties to implement a waste sorting policy and guided farmers to participate in this project in order to improve their ecological environment. Those Chinese farmers affected by this policy are actively participating in waste sorting activities. The government and relevant departments put up posters, handed out leaflets, and broadcast knowledge related to garbage classification. Farmers not only actively learned the knowledge related to waste sorting, but also received and used some waste sorting equipment provided by the government, such as trash cans.

Domestic waste sorting is an effective method for solving waste disposal problems and improving the quality of the living environment [2]. Many countries have also implemented a rural domestic waste sorting system. For example, in EU countries, farmers put differently classified garbage into different-colored trash bins, and if the farmers’ garbage is classified incorrectly, the garbage collector will refuse to collect it, or may even fine the farmer [3]. In Japan, garbage is classified into combustible garbage, non-combustible garbage, resource garbage, and bulky garbage. Farmers put garbage in designated locations, and there are special personnel to collect the garbage regularly [4]. Due to the scattered residence of farmers in the United States, enterprises are responsible for the collection, transportation, and disposal of household waste after classification, and waste disposal fees are collected from farmers [5].

Early studies focused on the influencing factors affecting farmers’ participation in waste sorting [6,7,8,9,10], such as value perception [11], values [12], consumer awareness [13], social pressure [14], emotions [5], green information [15]. In recent years, some researchers have focused on the spillover effect of the waste sorting behavior (WSB), Xu (2018) [16] found that WSB has a spillover effect on urban household electricity consumption. Some studies used experimental methods to analyze whether the option of recycling increases resource consumption. Specifically, their first experiment showed that consumers used more paper when evaluating a pair of scissors if the option to recycle was provided (vs. if it was not provided). In a follow-up field experiment, they found that the per person restroom paper hand towel usage increased after the introduction of a recycling bin compared to when a recycling option was not available [17]. Some researchers have noticed that waste sorting leads to lower support for green funds [18], and people actively participating in waste sorting are less wasteful of resources in daily life [19].

The aforementioned studies show that WSB has an effect on pro-environment behavior, such as green consumption behavior, but there is still a lack of evaluation of the influence of WSB on green consumption behavior. Few researchers have focused on whether the participation of rural households in waste sorting activities could result in some spillover effect, especially with respect to green consumption behavior in developing countries or in rural area in developing countries. Green consumption is a moral behavior and has been recognized and promoted as a reform of the traditional consumption model [20]. It is strongly advocated in contemporary society [21], and both the government and businesses are taking various measures to encourage people to join in and promote green consumption [22]. Green consumption could not only force companies to produce environmentally friendly green products, accelerating the green reform of the supply side, but could also enhance the sense of acquisition, security, and happiness of residents [23].

In fact, the educational level of Chinese farmers is generally low, and their awareness of environment protection is weak [2]. Therefore, we wanted to know whether farmers’ participation in waste sorting increases their environmental concern and then promotes green consumption behaviors. Hence, the purpose of the study was to test the spillover effect of waste sorting behavior on green consumption and its mechanism of influence on the mediation effect of environmental concern. Thereby, we explain the theoretical mechanism of the impact of waste sorting behavior on rural household green consumption behavior. Then, based on field survey data from 688 rural households in the Shaanxi Province, China, firstly, a Logit model was employed to investigate the influencing factors on rural household participation in waste sorting behavior. In addition, then, a propensity score matching model was used to explore the impact of the spillover effect. Finally, the bootstrap mediating model was examined to empirically test the internal relationship between waste sorting, environmental concern, and rural household green consumption behavior. The results of this research will be helpful for broadening the research on the effects of waste sorting on farmers’ welfare. It may also provide some policy implications for further encouraging waste sorting, increasing farmers’ ecological concern, and promoting rural consumption transformation and development.

Our contribution to the existing literature is as follows. On the one hand, this article innovatively studies the spillover effect of waste sorting from the perspective of farmers’ green consumption behavior, which not only enriches the research on the green consumption behavior of residents, but also extends the welfare effects of waste sorting behavior. On the other hand, we investigated the impact mechanism of waste sorting and green consumption behavior. Specifically, we tested whether environmental concern played a mediating role between waste sorting and green consumption. Additionally, in terms of the research area, few studies have focused on the issues of waste sorting and green consumption in developing countries, especially in China, which ranks among the top in the world in terms of population.

## 2. Literature Review and Hypothesis Development

### 2.1. Analysis of the Relationship between Waste Sorting Behavior and Green Consumption Behavior

Waste sorting, resource recycling and consumption have become a hot topic of debate for maintaining environmental sustainability [24,25,26]. Many researchers have already conducted studies relevant to waste sorting and consumption. One study investigated the intensification of consumer waste sorting using a behavioral model in the existing literature and revealed that advertising the waste sorting policy made a great impression on consumers [27]. Consumer behavior regarding waste sorting can be affected through feelings and attitudes with respect to consumer preference and green consumption [28]. Another study investigated household waste sorting behavior and engagement in different life activities and concluded that the current waste sorting initiatives contributed to households attempting to pursue green consumption [29]. Environmental sustainability and sustainable production are directly connected with the household’s consumption behavior, and whether it is willing to pursue greener consumption. The evolving concept of green consumption is heavily affected by consumer beliefs, norms and preferences, but which is also, nevertheless, extremely nuanced, dynamic and context-dependent [30]. It has also been argued that, in the systematic conceptualization of environmental sustainability, green consumption must be considered within the discussion of healthy nutrition and a healthy environment [31]. Changes in household consumption behavior lead to significant changes in household waste, which is largely dependent on product packaging. There are also several factors that influence household waste behavior [14,32,33], including the 3Rs (reduce, reuse, recycle), societal infrastructure, legislation relating to waste sorting methods, product packaging, trends for household consumption, and income. The waste, for the purposes of waste sorting, can be divided into two groups: organic and inorganic. Organic waste includes animal waste, plants, discarded food, and garden accoutrements. Inorganic waste includes man-made products such as plastic, metal, and glass. Therefore, this study formulates the following hypothesis.

**Hypothesis 1** **(H1).** *Waste sorting behavior has a significantly positive impact on rural households’ green consumption.*

### 2.2. Mediation Effects of Environmental Concern

Many researchers believe that perception of the environment and psychological factors are important incentives affecting green consumption [34,35,36]. The theory of rational behavior suggests that people first form attitudes relating to their beliefs in things, then attitudes regarding intended behavior, and then individuals take actual actions based on those behavior intentions [37,38]. The theory of planned behavior thus believes that behavioral intention is the most direct psychological factor affecting the actual behavior of individuals [39]. This means that the more concerned residents are about environmental issues, the easier it is for them to form positive environmental awareness, and the more likely they are to increase green consumption in their daily lives [40].

A representative variable for environmental attitude is environmental concern [41,42,43], which refers to people’s degree of awareness and support for solving problems involving the ecological environment or their willingness to contribute to solving such problems [44,45,46]. Waste sorting significantly enhances the individual’s perceived value of and the concern about the environment [47]. The results of an empirical study revealed that a high level of environmental concern indicates a higher degree of consumer awareness of the environment and willingness to purchase green products, and the increase in green consumption was highly significant [48]. As noted, the literature indicates that environmental concern plays a mediating role in the impact of waste sorting on rural household green consumption. We thus propose the following hypothesis:

**Hypothesis 2** **(H2).** *Waste sorting behavior positively affects rural household green consumption through the indirect path of environmental concern.*

## 3. Materials and Methods

### 3.1. Data

**Figure 1:**
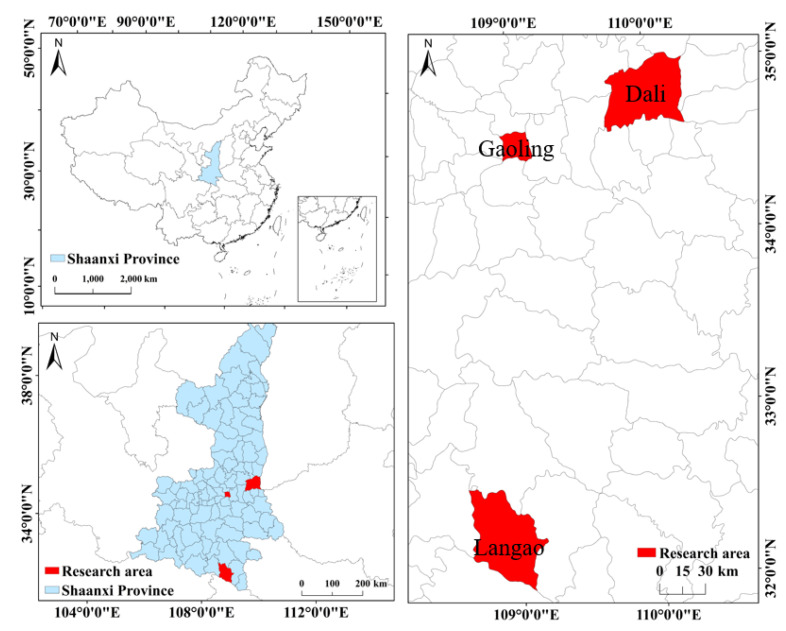
Map of the study area.

The Ministry of Housing and Construction of China chose 100 pilot counties to implement waste sorting in 2017. Four of those counties were in Shaanxi. Shaanxi is an important western agricultural area, and has a population of more than 20 million, approximately 46% of whom live in rural areas. Hence the sample of rural people of Shaanxi is representative of western China. A large-scale survey was carried out in the Shaanxi Province over the period 14–28 April 2018 (Figure 1). Considering the geographical location, economic development level, and the population distribution of the pilot areas, we chose three pilot counties: Gaoling District of Xi’an, Dali County of Weinan, and Langao County of Ankang. In total, 10 administrative villages from south to central Shaanxi were selected, and 2–3 sample villages (nature villages) were selected in each administrative village jointly considering distribution and levels of economic development. According to the principle of random stratified sampling, 20–25 farmers were randomly selected for interview in each sample village. Before the survey, we conducted one-on-one training with 15 PhD and master students around the questionnaire questions to ensure that they had a consistent understanding of the questionnaire content and the same inquiries. Then, taking into account the low education levels of the rural people in China, the students conducted face-to-face interviews with farmers to ensure the validity of the questionnaires, with each interview lasting about one hour to ensure that every respondent could understand the questions. In the end, we approached 700 households and managed to complete 688 questionnaires, for a 98.28% response rate.

### 3.2. Variables

#### 3.2.1. Dependent Variable

Green consumption behavior means paying more attention to resource conservation, environmental friendliness and public health, and the consumption behavior should be harmonious and sustainable [49,50]. It is generally associated with purchasing in a responsible, ethical, sustainable, and environmentally friendly way [51]. It includes purchasing and using environmentally friendly products [52]. Researchers have characterized green consumption behavior on the basis of the following items: whether you often buy fruits and vegetables that have not been used with fertilizers and pesticides; whether you often deliberately reduce the consumption of oil, gas, electricity and other energy or fuel at home in order to protect the environment; you often avoid buying certain products specifically for environmental protection; whether you buy energy-saving products; whether you bring your own environmentally friendly shopping bags when shopping; and whether you buy products that contribute to health and buy products with environmentally friendly packaging [53,54].

Green consumption behavior was characterized as a dependent variable. This paper defines green consumption behavior as rural household’s choosing environmentally friendly products and protecting the environment when they consume and use products. This article used the factor analysis method to measure green consumption behavior according to six questions. We asked respondents how often they participated in the following activities. The options were represented using a five-point Likert scale, with values from 1 to 5 meaning very infrequently, relatively infrequently, general, relatively frequently and very frequently, respectively. The six questions were: (1) I purchase vegetables without chemical fertilizers and pesticides; (2) I buy energy-saving electronics; (3) I save electricity and water in daily life; (4) I bring my own reusable eco-friendly shopping bag or buy a biodegradable shopping bag; (5) I purchase fruits without chemical fertilizers and pesticides; and (6) I buy meat without veterinary drugs regularly.

The results of the correlation analysis of variables show that the correlation coefficient value of multiple variables is relatively high, which indicates that there is a significant correlation between these variables, and further illustrates the necessity of factor analysis. According to the requirement that the feature root is greater than 1, two common factors were extracted; the cumulative variance contribution rate was 62.451%. The Cronbach coefficient was used in the reliability analysis. The Cronbach’s α value of the green consumption behavior exceeded 0.8, which is in excess of the recommended value of 0.7 [55]. This suggests that reliability analysis is at an acceptable level. In addition, the values of factor loading of the variables exceed the 0.5 [56]; the value of KMO (Kaiser–Meyer–Olkin) was 0.738, which is in excess of 0.6; Bartlett’s test was significant (*p* < 0.01). Generally speaking, the indicators of variables were highly reliable and had good validity. The details of indicators are shown in Appendix A.

#### 3.2.2. Independent Variables

Considering the measurement value of farmers’ participation in WSB as a binary variable with participation or not, on the basis of related research [57,58], we set the core independent variable WSB as a binary variable. Additionally, studies have shown that individual, family, and regional characteristics such as age [59], the area where the sample farmers are located [60], age, education level, income level, occupation, family structure [61] significantly affect farmers’ WSB and green consumption behavior. 

Hence, we set WSB as the core independent variable, and characterized personal characteristics (householder age, householder age squared, householder political status, householder education years, householder education years squared); family characteristics (family size, household total income); and regional characteristics (whether the interviewed area is in Ankang, Xi’an) as control variables.

Specifically, to investigate WSB, we set the question “Whether you participate in WSB”. When the respondent replied “yes”, that meant the value of WSB was equal to 1. Otherwise, the value of WSB is equal to 0. “Hhead_age” refers to the householders’ age. “Hhead_political status” represents the political status of the head of household, and we set the question “whether householder is a party member”, when the respondent replied “yes”, that meant the value of it was equal to 1. Otherwise, the value was equal to 0. “Hhead_edu_years” refers to the length of education of the householder. “House_member_size” reflects the population size of the family. “Household total income” reflects the family’s income, and we adopted the following principle when calculating it: adding 1 to the income, and then take the natural logarithm. “Located in Ankang city” and “Located in Xi’an city” represent whether the interviewed area is in Ankang city or Xi’an city.

#### 3.2.3. Mediating Variable

Behavioral intention is the most direct psychological factor affecting the actual behavior of an individual [39]. This shows that when residents pay more attention to environmental issues, they form a more positive environmental awareness, thereby promoting their green consumption behavior [40]. Researchers generally believe that environmental concern can be used to characterize environmental attitudes [41,42,43]. This pertains to the extent to which consumers are cognizant of environment-related issues, and showcase the desire or inclination to participate in solving them [45]. In addition, it also reflects consumers’ positive feeling towards green issues and evaluation of the impact of individual activities on the environment [62,63]. Chuah et al. (2020) used the following four questions to measure environmental concerns: I am concerned about the environment; The condition of the environment affects the quality of my life; I am willing to make sacrifices to protect the environment; I am emotionally involved in environmental protection issues [64].

Environmental concern is the mediating variable. We used factor analysis to measure environmental concern according to four items: (1) Are you concerned about the air quality in your village; (2) Are you concerned about how others sort waste; (3) Are you concerned about the safety of drinking water in the village; and (4) Are you concerned about whether the food you eat every day is contaminated with pesticides and fertilizers. Responses were measured according to a 5-point Likert scale (completely disagree = 1, comparatively disagree = 2, generally = 3, comparatively agree = 4, completely agree = 5). After factor analysis, one common factor was extracted, with a cumulative variance contribution rate of 66.611%. The KMO value was 0.781, the significance of the Bartlett sphericity test statistic was 0.000, the Cronbach coefficients were all higher than 0.7, and the factor load values were all greater than 0.5, showing that all the values of the correlation, reliability, and convergence validity are at an accepted level indicating the validity of factor analysis.

The definition and descriptive statistics of the above variables are shown in Table 1.

### 3.3. Methodology

According to the random utility decision model, variable $U_{1i}$ and variable $U_{0i}$ can be set to represent the utility of farmer i participating or not participating in waste sorting, and $M_{i}{}^{*}$ is defined as the difference between the two—that is, $M_{i}{}^{*}=U_{1i}-U_{0i}$. Microeconomics assumes that individuals are completely rational and pursue maximum utility; if $M_{i}{}^{*}>0$, it means that the farmer will participate in waste sorting, that is $M_{i}{}^{*}=1$; otherwise, it means that the farmer will not participate in waste sorting, that is $M_{i}{}^{*}=0$.

This paper sets the equation for participating in waste sorting as:

(1) $$M_{i}{}^{*}=\psi (x)+\varepsilon$$

In Equation (1), $M_{i}{}^{*}$ is a binary explanatory variable; x indicates the exogenous explanatory variables that affect farmers’ participation in waste sorting, including personal characteristics, family characteristics and regional characteristics (specific variables shown in Table 1); and ε represents random error.

To measure the effect of waste sorting on rural household green consumption, we set the equation for rural household green consumption as:

(2) $$Y_{i}{}^{*}=\phi (Z)+\lambda M_{i}+\delta$$

In Equation (2), $Y_{i}{}^{*}$ is the latent variable of farm household green consumption; Z indicates the exogenous explanatory variables that effect rural household green consumption; $M_{i}$ is the variable of farmer i participating in waste sorting; and δ is the random disturbance term. Because whether a farmer chooses to participate in waste sorting ($M_{i}$) may be affected by certain unobservable factors—which may be related to rural household green consumption ($Y_{i}{}^{*}$), which in turn leads to the correlation between $M_{i}$ and δ the measurement results may be biased if the regression analysis is performed directly.

Compared with traditional linear regression, propensity score matching can effectively overcome biased estimation and the selection bias caused by sample self-selection, and there are obvious advantages in solving the endogenous problem of variables [65]. We therefore chose to use this method to estimate the impact of waste sorting on rural household green consumption.

With reference to the classic counterfactual analysis framework, this paper sets a dummy variable $D_{i}=\{0,1\}$ to indicate whether the farmer i participates in waste sorting: i=1 means yes, and i=0 means no. For individual i, the future household green consumption $y_{i}$ may have two states: $y_{1i}$ represents the future household green consumption of farmer i participating in waste sorting, and $y_{0i}$ represents the future household green consumption of farmer i not participating in waste sorting. The research steps for the counterfactual analysis framework in this paper are as follows:

The first step is selecting the covariate $x_{i}$. Drawing on the relevant literature, factors that affect rural household green consumption and waste sorting are included in the model to ensure that the negligibility assumption is satisfied.

The second step is calculating the propensity scores. In this paper, a Logit model is used to calculate the propensity scores of farmers who participate in waste sorting. Following the recommendations of Rosenbaum and Rubin [66], introducing the higher-order terms of $x_{i}$ in the model (such as households’ age squared, households’ education years squared) makes the equation more flexible, thereby further improving the accuracy of the calculation results.

The third step is to perform propensity score matching. First, we selected the matching methods. To enhance the reliability of the research conclusion, we used K-nearest neighbor matching method, nearest-neighbor matching with the calliper method, kernel matching method, and the spline matching method [67].

The fourth step is calculating the average treatment effect. There are three types of treatment effect. The first type is average treatment effect on the treated (ATT), which is the average value of green consumption changes among farmers who participate in waste sorting. The second type is average treatment effect on the untreated (ATU)—that is, the average change in green consumption among rural households who do not sort waste. The third type is the average treatment effect (ATE) of the whole sample, which is the average value of rural household green consumption changes for random samples. Because this article explores the impact of waste sorting on rural household green consumption and we pay more attention to changes in household green consumption among farmers participating in waste sorting, it is more appropriate to use ATT for analysis. The formula is as follows:

(3) $$\hat{ATT}=\frac{1}{N_{1}}\sum_{i:D_{i}=1}\left(y_{i}-{\hat{y}}_{0i}\right)$$

In Equation (3), $N_{1}$ represents the number of farmers in the treatment group, which are the number of farmers participating in waste sorting; $\sum_{i:D_{i}=1}$ adds up the farmers who participate in waste sorting; $y_{i}$ represents the green consumption of farmer i; and ${\hat{y}}_{0i}$ represents the estimated value of green consumption in rural households participating in waste sorting, assuming they did not participate in waste sorting.

## 4. Estimations and Results

### 4.1. Influencing Factors on Waste Sorting Behavior

To ensure that the samples were effectively matched, we needed to analyze the factors affecting farmers’ participation in WSB. The estimated results are shown in Table 2. The Pearson test results for the correlation between independent variables show that there is no multicollinearity between the variables. It can be seen from Table 2 that personal characteristics, family characteristics and regional characteristics are important incentives for farmers to participate in WSB.

From the perspective of personal characteristics, “Hhead_age”, “Hhead_age squared”, “Hhead_political status”, “Hhead_edu_years” affect WSB significantly. This means that householder age, householder political status and householder education years have significant positive impacts on farmers’ participation in WSB. This conclusion is consistent with [59,60,61]. As the age of the head of the household increases, there is greater willingness to participate in WSB. Additionally, the effect of “Hhead_age squared” on WSB is −0.001, and it is significant at a statistical level of 1%. This indicates that householder age has a “Reverse U” relationship with such participation, meaning, compared with young and old householders, and middle-aged householders are more likely to participate in WSB. Furthermore, the effect of “Hhead_political status” was 0.806, and is significant at a statistical level of 5%. This indicates that if householders are party members, they have an especially strong tendency to participate in WSB.

From the perspective of family characteristics, “House_member_size” and “Household total income” significantly affected WSB at a statistical level of 1%, and the effects of “House_member_size”, and “Household total income” were 0.770 and 0.251, respectively. This means that family members and total household income could promote farmers’ participation in WSB effectively. When family members or income increase by one unit, the probability of farmers participating in WSB increases by 77% and 25.1%.

From the perspective of regional characteristics, “Located in Xi’an city” had a significant positive impact at the 1% level, and its effect was 0.672. The possible reason for this is that Xi’an city is the capital city of Shaanxi Province. It has good performance on an economic level, and the GDP keeps ranking at the top of Shaanxi Province. Good economic conditions lead to better support for waste sorting policies. The local governments and relevant departments with better conditions are ablet to provide farmers with more sorting equipment, such as trash cans, which can be helpful in promoting farmers’ participation in WSB.

### 4.2. The Effect of Waste Sorting Behavior on Green Consumption

Before starting to use the propensity score matching (PSM) method for empirical analysis, we need to judge whether the PSM method is effective. This needs to be tested with respect to the following three aspects.

**Figure 2:**
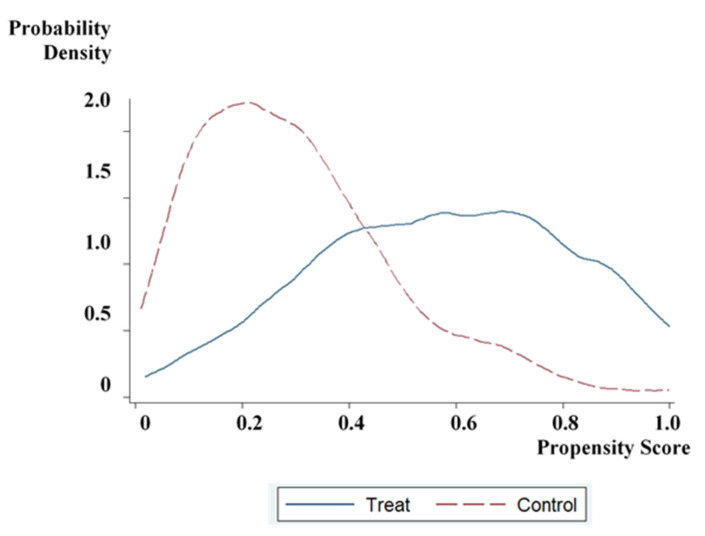
Density function graph before propensity score matching.

**Figure 3:**
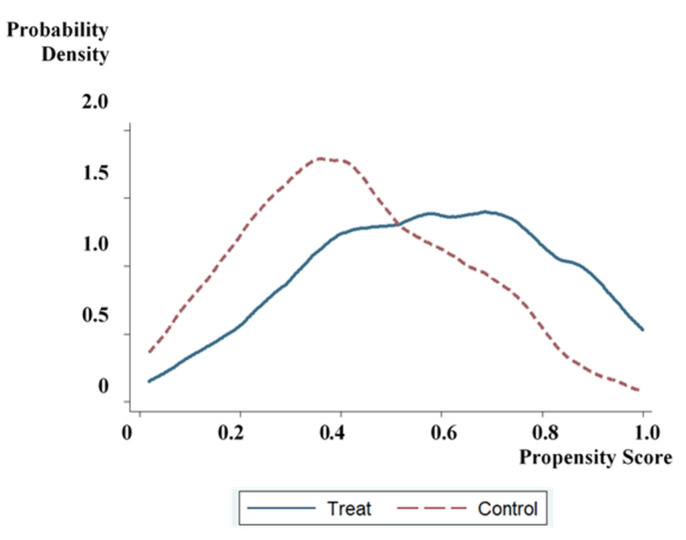
Density function graph after propensity score matching.

On the one hand, to ensure the matching quality of the sample data, we drew two density function graphs to check the common support domain before and after matching, as shown in Figure 2 and Figure 3. The tendency scores of the participating samples and the non-participating samples have a large overlap, and most of the observations are within the common value range.

On the other hand, according to the maximum loss results of the samples under the four different matching methods, although three samples were lost, the treatment group and the control group still retained 685 matching samples, indicating that the matching effect was good.

In addition, we examined the balance of the covariates to ensure the reliability of the matching results for the propensity score. If the data pass the balance test, there will be no significant systemic difference in covariates between the treatment group and the control group after matching except for differences in rural household green consumption. From the balance test results (as shown in Table 3), after matching, the standardized bias was reduced from 36.1% to between 9.4% and 11.9%; the total bias is significantly reduced and it is less than the 20% standard prescribed by the balance test; the Pseudo R^2^ dropped from 0.200 to between 0.023 and 0.026; and the LR statistics decreased from 187.5 to between 17.93 and 20.86. According to the analysis of the above test results, using propensity score matching can effectively reduce the differences in the distribution of explanatory variables and eliminate estimation bias caused by self-selection.

The above test results confirm the effectiveness of the PSM method, so we can use this method to analyze the average treatment effect of WSB on farmers’ green consumption behavior. The estimation results of the four different matching methods are basically consistent indicating a good robustness of the data (as shown in Table 4). We therefore calculate its arithmetic mean to measure the effect value.

Based on the counterfactual estimation of PSM, WSB positively and significantly affected rural household green consumption behavior, with a net effect of 0.205. This indicates that participation in waste sorting activities will promote a significant increase of 20.5% of rural household green consumption after considering the selection bias. Hypothesis H1 is thus verified. This conclusion is consistent with Xu et al. [68] and Peattie [30]. The possible reasons for this are that, in China, most rural people are not well educated, leading to low awareness and behavior with respect to environmental protection. The Chinese government has recently promoted the construction of beautiful villages, rural households have begun to participate in WSB, and they will experience the environmental changes and the improvement in their lives. Hence, they are willing to develop green consumption behavior, such as purchasing vegetables and fruits without chemical fertilizers and pesticides, buying meat without veterinary drugs, bringing their own reusable eco-friendly shopping bags or buying biodegradable shopping bags, buying energy-saving electronics, and saving electricity and water in daily life.

### 4.3. The Mediating Effect of Environmental Concern

The mediating effect model was used to explore the mechanism of influence of WSB on green consumption behavior. Currently, there are two main methods for testing mediation. The first is the Sobel test, advocated by Baron and Kenny [69], but this method has obvious defects. The standard error of its hypothesis test is obtained by unbiased or biased estimation. To overcome these problems and more accurately verify the mediating role of environmental concern in the impact of WSB on rural household green consumption behavior, we used the Bootstrap method for testing. This method repeats sampling from the samples. If 0 is not within the upper and lower limits, it indicates that the mediating effect is significant within the confidence interval [70,71].

The results of the mediating test (as shown in Table 5) show that under the 95% confidence interval, LLCI (Lower Level of Confidence Interval) is 0.2405, ULCI (Upper Level of Confidence Interval) is 0.4125 and 0 is not included, which indicates that the mediating effect (0.3177) of environmental concern is significant. After controlling for environmental concern, the impact of WSB on rural household green consumption behavior is still significant, LLCI is 0.3156, ULCI is 0.0549, and 0 is not included, indicating that environmental concern plays a mediating role in the relationship between WSB and rural household green consumption behavior. Thus, hypothesis H2 is verified. This finding is consistent with extant research [34,35,36,40].

The possible reason for this is that local government carried out a large number of policies to encourage more farmers to participate in WSB in China. In the process of farmers’ participation in WSB, they realized the importance of WSB and the method of garbage classification, thus improving their awareness of environmental protection and environmental concern. When households have environmental consciousness, they are more likely to participate in pro-environmental behavior. Consequently, WSB positively affects rural household green consumption behavior through the indirect path of environmental concern. In summary, the results further indicated that WSB not only impacted rural household green consumption behavior directly, but also impacted it indirectly via environmental concern.

## 5. Conclusions, Policy Implication and Research Limitations

### 5.1. Conclusions

The purpose of this paper was to explore the influence of WSB on green consumption behavior and possible psychological mechanisms of mediation of environmental concern. The study used field survey data from 688 rural households from three cities and three counties in the Shaanxi Province in western China. The main conclusions are as follows.

Firstly, personal characteristics, family characteristics and regional characteristics are important factors influencing farmers’ WSB. Specifically, householder age has a “Reverse U” relationship with WSB. This means that compared to young and old farmers, middle-aged people are most likely to participate in WSB. If householders are a party member and mature, they are more willing to participate in WSB. Additionally, family members and total household income significantly affect WSB. In addition, better regional economic conditions can provide more waste disposal facilities, including trash cans, etc., to promote farmers’ participation in WSB.

Secondly, WSB has a significant impact on the rural household green consumption behavior, with a net effect of 0.205. This means that when farmers participate in WSB, the possibility of their green consumption behavior can be increased 20.5%. The results indicated that rural households sort waste in their daily lives; thus, they are more inclined to participate in green consumption activities. The results are consistent with relevant research [24,26,29]. When the Chinese government implemented the waste sorting policy, farmers found that the living conditions of villages and waste management are improved. Therefore, they gradually realized the practical significance of environment protection caused by waste sorting policies. Simultaneously, they will be more proactive in participating in other pro-environment activities simultaneously, such as green consumption behavior. For example, they actively buy fruits and vegetables without fertilizers and pesticides, buy meat without veterinary drugs, and prefer to save water and electricity. All this shows that WSB has a positive impact on individuals’ green consumption behavior.

Furthermore, this study found a mediating effect of environmental concern on WSB affecting the green consumption behavior, with a mediating effect of 0.3177. This indicates that the farmers’ WSB indirectly promotes their green consumption behavior, with a value of 0.3177. This means that environmental concern has a partial mediating effect (indirect effect) in the impact of WSB on farmers’ green consumption behavior, with a degree of influence of 31.77%. This finding is consistent with previous research [34,35,36,40]. To examine the influencing mechanism, we employed a bootstrap mediating test model to examine the effect of environmental concern. The results show that environmental concern played a mediating role in the impact of WSB on rural household green consumption behavior. In the process of participating in garbage sorting activities, farmers acquired knowledge and methods of environmental protection from local governments and other institutions. For example, they understood the types of garbage, garbage disposal methods, the hazards of garbage, etc. After understanding content related to waste, their environmental responsibility is stronger, and their degree of environmental concern is also increased. This means that farmers experienced an increase in their environmental concern by participating in WSB, which then promoted their participation in green consumption behavior. This is the influencing mechanism of the spillover effect of waste sorting on pro-environment behaviors.

### 5.2. Policy Implications

This study on the spillover influence of household waste sorting on green consumption behavior by the mediation of environmental concern has some implications for governmental and pro-environmental agencies. The local government should expand more pilot areas to encourage more people to participate in waste sorting activities, which are helpful for improving rural households’ pro-environmental green consumption behavior, including buying environmentally and saving water and electricity. The countermeasures are as follows.

Firstly, local government should provide information online and offline describing the meaning, methods, and welfare effects of waste sorting in order to encourage rural households to join in. Additionally, they could also establish a comprehensive assessment standard for household waste sorting systems. In addition, they should also encourage rural residents to participate in waste sorting activities by both incentives policy for action and punishment policy for non-participation.

Secondly, it should take some measures to encourage people to develop green consumption behavior. For example, policy could assist rural households in understanding the concept and meaning of green consumption, and then stimulate them to regularly purchase sustainable and environmentally friendly products, such as vegetables, fruits without chemical fertilizers and pesticides, and to buy meat without veterinary drugs. Additionally, the government also needs to provide corresponding economic construction and vigorously develop the economy, so that it can increase residents’ income, which would be helpful for breaking people away from lower-level consumption patterns. This would be helpful for farmers in meeting the standards of basic production and living.

Finally, the government should pay more attention to the education and cultivation of rural households’ environmental concern. Specifically, the government should increase the publicity of waste sorting activities, continuously improve the environmental concern of rural households, increase farmers’ internalization and environmental protection awareness, and transform green consumption concepts into green consumption behaviors.

### 5.3. Research Limitations

This research is helpful for understanding the spillover effect of household waste sorting on green consumption behavior and its mechanism. However, some limitations of this study should be pointed out. First, the data were collected in Shaanxi Province. Although Shaanxi Province is a pilot province for waste sorting and shares some common characteristics with other provinces [19], rural households’ environmental concern and waste sorting behavior may be different from those in other provinces. Thus, generalization of the present study’s results to other research contexts should be done with caution. In future research, survey data should be collected from more pilot areas. Second, we analyzed the mediating role of environmental concern in this paper. However, other mediating variables still exist, such as income, perceived value, and so on. Hence, future research should consider more possible paths of influence between WSB and green consumption behavior. Third, limited control variables were added to the PSM model to explore waste sorting and green consumption behavior. Some variables, such as emotion, governmental action, are not considered. In the future, researchers can take these variables into account to extend the present research.

## Figures and Tables

**Table 1:** Variable definition and data description (*N* = 688).

Variables	Definition	Mean	Std
Green consumption behavior	Factor analysis	0.000	0.751
Waste sorting	Whether sorting waste, yes = 1, no = 0	0.417	0.493
Hhead_age	Householder age	48.087	10.201
Hhead_age squared	Householder age squared	2146.776	1151.958
Hhead_political status	Whether householder is a party member, yes = 1, no = 0	0.096	0.294
Hhead_edu_years	Householder education years	7.410	3.542
Hhead_edu_years squared	Householder education years squared	67.438	51.798
House_member_size	Family size	3.448	1.170
Household total income	Adding 1 to the Household total income, and then take the natural logarithm	11.666	1.248
Located in Ankang city	Whether the interviewed area is in Ankang city, yes = 1, no = 0	0.171	0.377
Located in Xi’an city	Whether the interviewed area is in Xi’an city, yes = 1, no = 1	0.323	0.468
Environmental concern	Factor analysis	−6.61e-07	0.999

**Table 2:** Estimation results for farmer participation in formal lending equation based on the Logit model.

Variables	Regression Coefficient	Standard Error
Hhead_age	0.057 ***	0.019
Hhead_age squared	−0.001 ***	0.000
Hhead_political status	0.806 **	0.324
Hhead_edu_years	0.153 *	0.081
Hhead_edu_years squared	−0.008	0.005
House_member_size	0.770 ***	0.109
Household total income	0.251 ***	0.085
Located in Ankang city	0.264	0.371
Located in Xi’an city	0.672 ***	0.264
Log likelihood	−366.918	
R^2^	0.217	
Likelihood ratio chi^2^	203.790	
Observations	688	

Note: *, **, and *** denote a statistical significance at the 10%, 5%, 1% level, respectively.

**Table 3:** Balance test results before and after matching.

Matching Methods	Pseudo R^2^	LR chi^2^	MeanBia (%)
Before matching	0.200	187.500	36.100
K-nearest neighbor matching method (k = 4)	0.026	20.860	10.600
Nearest-neighbor matching within caliper method (k = 4, cal = 0.063)	0.026	20.860	10.600
Kernel matching method	0.023	17.930	9.400
Spline matching method	0.026	20.800	11.900

**Table 4:** Impact of waste sorting on China rural household green consumption.

Matching Methods	Average Treatment effect on the Treated (ATT)	Standard Error	T-Statistics
K-nearest neighbor matching method (k = 4)	0.209 **	0.099	2.120
Nearest-neighbor matching within caliper method (k = 4, cal = 0.063)	0.120 **	0.113	2.120
Kernel matching method	0.261 ***	0.095	2.740
Spline matching method	0.230 **	0.108	2.140
Average value	0.205	-	-

Note: **, and *** donate a statistical significance at the 10%, 5%, 1% level, respectively.

**Table 5:** Mediating effect based on the bootstrap method.

Effect	Coefficient	Boot Standard Error	LLCI	ULCI
Indirect effect	0.3177	0.0437	0.2405	0.4125
Direct effect	0.1853	0.0664	0.3156	0.0549

Notes: Using the non-parametric percentile bootstrap method for deviation correction, the number of repeated samplings is 5000, with a 95% confidence interval. If the 95% confidence interval is selected, meaning, LLCI represents the 2.5th percentile and ULCI represents the 97.5th percentile.

## Appendix A

**Table A1:** Results of the correlation of the variables. KMO: Kaiser–Meyer–Olkin

Variables	I purchase Vegetables without Chemical Fertilizers and Pesticides.	I Buy Energy-Saving Electronics.	I Save Electricity and Water in Daily Life.	I Bring My Own Reusable Eco-Friendly Shopping Bag or Buy a Biodegradable Shopping Bag.	I Purchase Fruits without Chemical Fertilizers and Pesticides.	I Buy Meat without Veterinary Drugs Regularly.
I purchase vegetables without chemical fertilizers and pesticides.	1.000	0.045	0.009	0.019	0.086	0.111
I buy energy-saving electronics.	0.045	1.000	0.743	0.695	0.049	0.118
I save electricity and water in daily life.	0.009	0.743	1.000	0.786	0.058	0.182
I bring my own reusable eco-friendly shopping bag or buy a biodegradable shopping bag.	0.019	0.695	0.786	1.000	0.071	0.157
I purchase fruits without chemical fertilizers and pesticides.	0.086	0.049	0.058	0.071	1.000	0.172
I buy meat without veterinary drugs regularly.	0.111	0.118	0.182	0.157	0.172	1.000

**Table A2:** Factor analysis results of variables.

Factor	Variables	Factor Loading	KMO	Bartlett’s Test	Cronbach’s α
1	I purchase vegetables without chemical fertilizers and pesticides.	0.580	0.738	1322.648 (0.000)	0.810
	I purchase fruits without chemical fertilizers and pesticides.	0.682			
	I buy meat without veterinary drugs regularly.	0.663			
2	I bring my own reusable eco-friendly shopping bag or buy a biodegradable shopping bag.	0.906			
	I buy energy-saving electronics.	0.927			
	I save electricity and water in daily life.	0.887			
